# SWI/SNF Chromatin Remodelling Complex in Hepatic Physiology: Mechanistic Insights Into Development, Homeostasis and Pathogenesis

**DOI:** 10.1111/jcmm.71032

**Published:** 2026-01-23

**Authors:** Rui Li, Bo Zhang, Xinyu Wei, Jiayin Yang, Yongjie Zhou

**Affiliations:** ^1^ Department of Liver Transplantation Center, Institute of Organ Transplantation, Frontiers Science Center for Disease‐related Molecular Network, West China Hospital Sichuan University Chengdu Sichuan China; ^2^ Department of Critical Care Medicine, West China Hospital Sichuan University Chengdu Sichuan Province People's Republic of China; ^3^ Key Laboratory of Transplant Engineering and Immunology, NHC, West China Hospital Sichuan University Chengdu China

**Keywords:** BRG1, chromatin remodelling, HCC, liver diseases, SWI/SNF complex

## Abstract

The liver executes essential metabolic functions including energy homeostasis, lipid biosynthesis, cholesterol regulation and xenobiotic detoxification. While hepatocyte metabolic activity forms the foundation of these processes, their precise regulation is achieved through chromatin remodelling mechanisms, with the SWI/SNF complex emerging as a central epigenetic orchestrator. Accumulating evidence positions this ATP‐dependent chromatin remodeler as a critical regulator of hepatic development, homeostatic maintenance and pathological transformation. Through nucleosome repositioning and histone‐DNA interaction modulation, the SWI/SNF complex governs transcriptional programs controlling cellular proliferation, differentiation and metabolic adaptation. This review synthesises current understanding of SWI/SNF‐mediated epigenetic regulation in hepatic biology and explores its therapeutic potential for liver disorders.

**Abbreviations**
BAF: BRG1/BRM‐associated factor
cBAF: canonical BAF complex
CCA: cholangiocarcinoma
CCND1: cyclin D1
CCNE1: cyclin E1
CTL: cytotoxic T‐lymphocyte
ECM: extracellular matrix
FAO: fatty acid oxidation
GBC: gallbladder carcinoma
HBx: hepatitis B virus X protein
HCC: hepatocellular carcinoma
HPCs: hepatic progenitor cells
HSC: hepatic stellate cell
I/R: ischemia–reperfusion
iCCA: intrahepatic cholangiocarcinoma
MMP: mitochondrial membrane potential
NAFL: non‐alcoholic fatty liver
NAFLD: non‐alcoholic fatty liver disease
NASH: non‐alcoholic steatohepatitis
ncBAF: non‐canonical BAF complex
NER: nucleotide excision repair
Nrf2: nuclear factor erythroid 2‐related factor 2
OIS: oncogene‐induced senescence
OS: overall survival
PBAF: polybromo‐associated BAF complex
PCNA: proliferating cell nuclear Antigen
pH 3: phosphorylated histone H3
PH: partial hepatectomy
ROS: reactive oxygen species
SWI/SNF: SWItch/sucrose non‐fermentable complex
TILs: tumour‐infiltrating lymphocytes

## 1 Introduction

**FIGURE 1:**
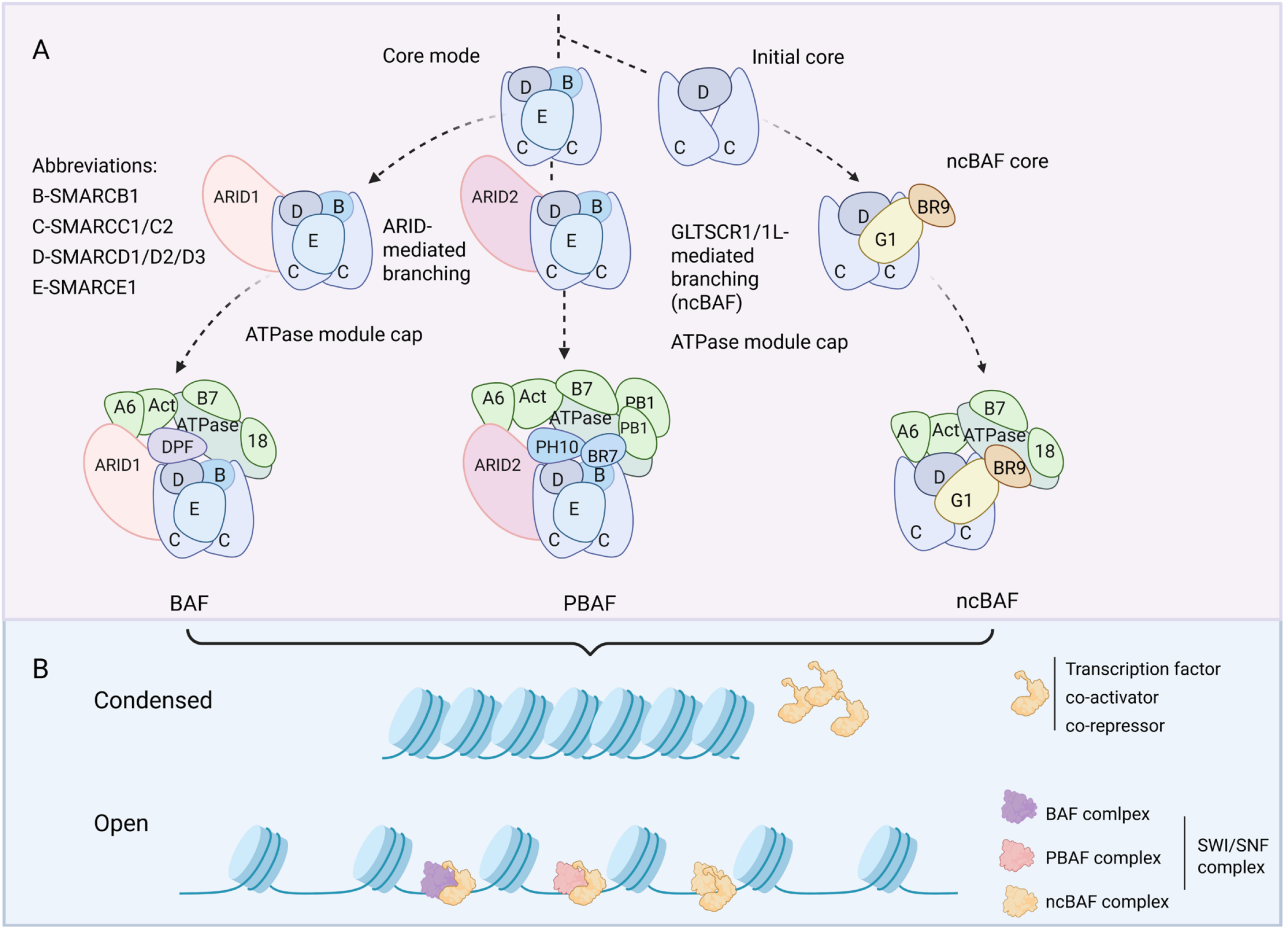
Assembly and functions of mammalian SWI/SNF complexes. (A) mSWI/SNF complexes assemble via a modular pathway. The process starts with a subcomplex of SMARCD and SMARCC, then incorporates SMARCE1 and SMARCB1. ARID subunits direct BAF/PBAF assembly, while GLTSCR1/1 L directs ncBAF assembly. Finally, the ATPase module completes the assembly. (B) The SWI/SNF complex loosens chromatin using ATP hydrolysis energy and regulates gene expression by interacting with transcription factors.

The SWI/SNF (SWItch/Sucrose Non‐Fermentable) chromatin remodelling complex, alternatively termed the BAF (BRG1/BRM‐associated factor) complex, represents an evolutionarily conserved family of ATP‐dependent chromatin remodelers critical for transcriptional regulation. These macromolecular assemblies utilise ATP hydrolysis to disrupt histone‐DNA interactions [1], enabling nucleosome repositioning, eviction, or histone variant exchange to modulate chromatin accessibility [2], originally identified in the yeast *Saccharomyces cerevisiae* for its role in *SUC2* gene activation and mating‐type switching [3, 4]. It typically consists of approximately 15 proteins, with component modifications depending on cellular conditions [5]. The mammalian SWI/SNF complex exists as three biochemically distinct isoforms: the canonical BAF complex, PBAF (polybromo‐associated BAF) and ncBAF (non‐canonical BAF) [6], as shown in Figure 1. Catalytic functionality is mediated by the mutually exclusive ATPase subunits SMARCA4 (BRG1) and SMARCA2 (BRM) [7, 8], which exhibit both divergent and synergistic functions across cellular contexts [9, 10]. Structural integrity is maintained by core subunits including SMARCB1 (SNF5), SMARCC1 (BAF155) and SMARCC2 (BAF170) [11, 12], while accessory components such as ACTL6A (BAF53) and SMARCE1 (BAF57) confer target specificity through transcription factor interactions (Figure 1) [5, 13]. This versatile complex orchestrates diverse biological processes including transcriptional reprogramming, DNA damage response and lineage specification, with particular functional complexity observed in mammalian systems [14].

As the principal metabolic organ, the liver coordinates glucose homeostasis, lipid biosynthesis, xenobiotic detoxification and immunomodulation [15]. The precise execution of these functions requires dynamic chromatin restructuring, exemplified by BRG1‐mediated regulation of hepatocyte metabolic programs [16]. Emerging evidence implicates both structural core subunits (e.g., SNF5) and regulatory components (e.g., BAF60 paralogs) in hepatic transcriptional networks through signalling pathway integration and epigenetic modulation [17, 18]. Crucially, SWI/SNF dysregulation manifests clinically through altered gene expression profiles that drive hepatic pathophysiology. In hepatocellular carcinoma (HCC), subunit‐specific perturbations demonstrate oncogenic consequences: ARID1A (BAF250a) inactivation promotes metastatic progression via steatosis‐associated genomic instability [19]. BRG1 overexpression strongly correlates with invasive phenotypes of HCC [20], while BRM downregulation associates with tumour differentiation status [21]. And BAF250a deletion in cholangiocarcinoma (CCA) enhances ALDH1A1‐mediated cancer stem cell expansion [22]. These findings collectively establish SWI/SNF components as nodal regulators of hepatic pathophysiology.

Recent advances in epigenomic profiling have substantially expanded our understanding of SWI/SNF‐mediated transcriptional control in hepatic physiology. However, critical knowledge gaps persist regarding subunit‐specific regulatory hierarchies, post‐translational modifications governing complex assembly and temporal–spatial functional dynamics during disease progression. This review systematically synthesises current mechanistic insights into SWI/SNF‐mediated chromatin remodelling in hepatic development, metabolic homeostasis and oncogenic transformation, while proposing novel therapeutic strategies targeting context‐specific complex functionalities.

## 2 SWI/SNF Complex Orchestrates Hepatic Morphogenesis Through Epigenetic Regulation of Terminal Differentiation

Hepatic morphogenesis initiates during embryonic development through endodermal differentiation into hepatocytes [23], and intercellular junctions are necessary for terminal hepatocyte differentiation [24]. Critical to this process, the SWI/SNF subunit SNF5 (encoded by *SMARCB1*) regulates approximately 70% of developmentally activated hepatic genes. Through a combination of ultrastructural analysis and transcriptomic profiling, Gresh et al. demonstrated that SNF5 deficiency precipitates a systemic failure in intercellular connectivity, characterised by widened intermembrane spaces and disrupted junctional complexes. The Zonula occludens 1 (ZO‐1) protein is normally localised to tight junctions; E‐cadherin is also involved in cell–cell interactions and is normally localised at both adherens junctions and desmosomes [25, 26]. Specifically, immunohistochemical assessments revealed a near‐complete absence of the tight junction marker ZO‐1 and markedly reduced expression of E‐cadherin at adherens junctions in mutant hepatocytes. These molecular and structural defects are further accompanied by cytoplasmic abnormalities featuring significantly reduced organelle and mitochondrial content, providing ultrastructural evidence of arrested terminal differentiation [17]. However, 30% of liver‐specific and developmentally activated genes are expressed at normal levels in mutant livers, indicating that the absence of SNF5 does not completely block hepatocyte differentiation. The normal persistence of these gene transcription rates may be due to residual SWI/SNF activity in inactivated cells. A study indicates that SNF5 inactivation is insufficient to disrupt all BRM‐ and BRG1‐associated activities [27].

Notably, SNF5 inactivation paradoxically enhances hepatocyte proliferation despite the characteristic association of differentiation with cell cycle exit via p21 upregulation [17]. This aberrant proliferative phenotype likely reflects compromised differentiation programming, as evidenced by coordinated downregulation of junctional proteins and cell cycle checkpoint bypass. Collectively, these findings establish the SWI/SNF complex as a master epigenetic regulator of hepatocyte maturation, integrating chromatin remodelling with structural and functional specialisation during liver development.

## 3 SWI/SNF Complex Maintains Liver Function and Homeostasis

### 3.1 Regulation of Lipid Metabolism

**FIGURE 2:**
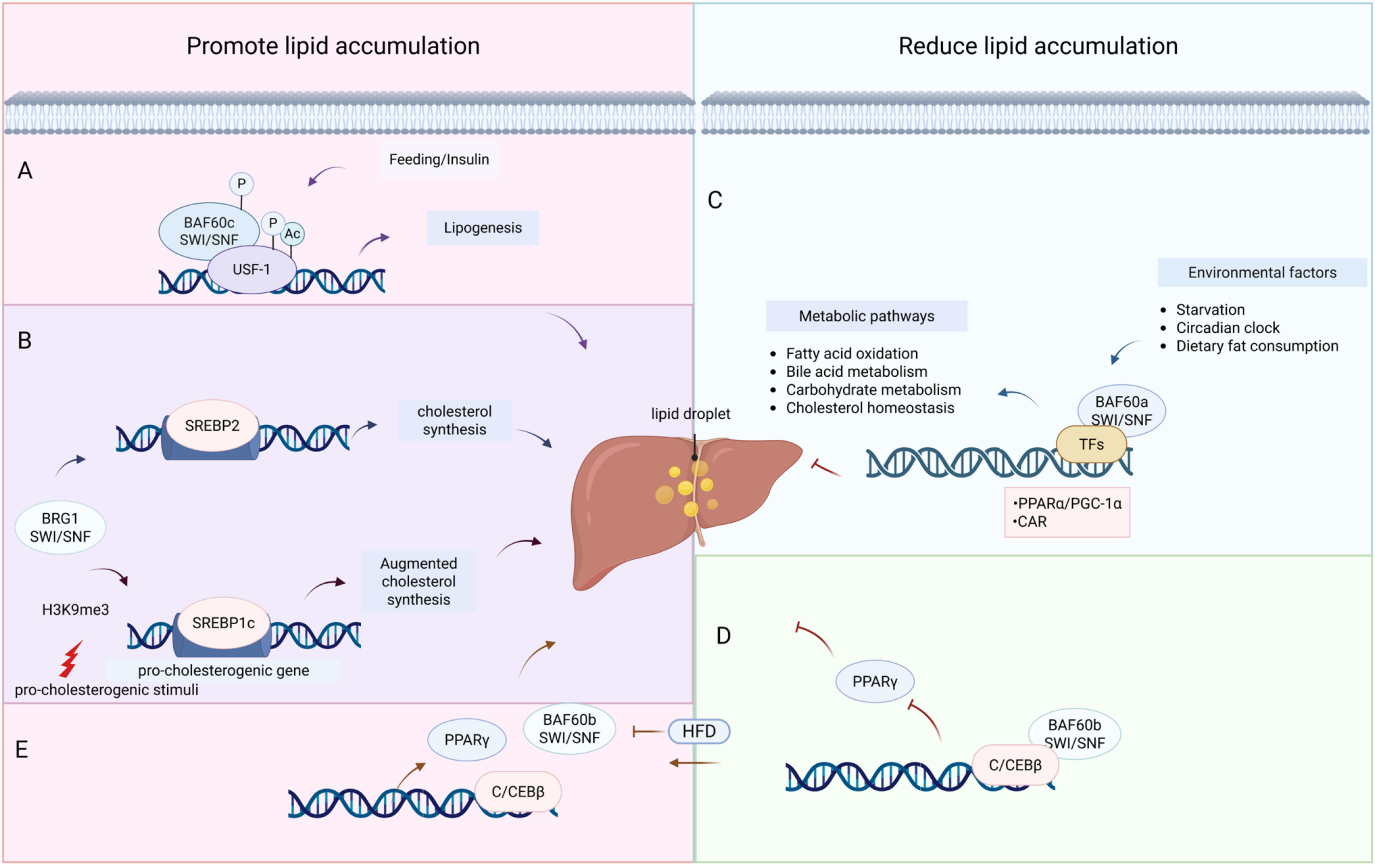
The three isoforms of the BAF60 subunits (BAF60a, BAF60b and BAF60c) and BRG1 regulate lipid metabolism. (A) Post‐feeding/insulin, phosphorylated BAF60c translocates to the nucleus, recruits SWI/SNF via phosphorylated/acetylated USF‐1 to activate lipogenic genes. (B) SREBP2/1c recruits BRG1 to promoters of cholesterol biosynthesis/fatty acid esterification genes. (C) BAF60a activates metabolic pathways through transcription factors (TFs)/cofactors interactions and SWI/SNF remodelling, regulated by fasting/circadian rhythm/diet. (D) BAF60b binds C/EBPβ to suppress PPARγ. (E) High‐fat diet downregulates BAF60b, upregulating PPARγ to promote lipogenesis. Ac, acetylation; P, phosphorylation; TFs, transcription factors; USF‐1, upstream stimulatory factor 1.

The liver centrally regulates systemic lipid metabolism through coordinated control of fatty acid oxidation, lipogenesis and cholesterol homeostasis [28]. Within the SWI/SNF complex, the BAF60 paralogs (BAF60a, BAF60b, BAF60c) serve as nutrient‐sensitive epigenetic regulators that integrate metabolic signals with chromatin remodelling (Figure 2) [29, 30, 31]. BAF60a directly activates hepatic β‐oxidation by recruiting the SWI/SNF complex to PPARα‐targeted loci, with fasting‐induced BAF60a upregulation enhancing chromatin accessibility at fatty acid oxidation (FAO) genes (e.g., *PGC‐1α*, *PPARα*) [32, 33]. Conversely, hepatocyte‐specific BAF60a ablation disrupts FAO, precipitating steatosis and impairing CAR‐dependent cholesterol efflux, thereby conferring resistance to diet‐induced atherogenesis [30].

BAF60c exhibits insulin‐responsive adipogenic activity through PPARγ coactivation, where its ligand‐dependent interaction with the receptor's C‐terminal domain facilitates chromatin remodelling at lipogenic loci [34, 35]. In contrast, BAF60b suppresses PPARγ via C/EBPβ sequestration, functioning as a lipid‐sensing brake on hepatic lipogenesis. However, non‐alcoholic fatty liver disease (NAFLD) may serve as a contributing factor to the downregulation of C/EBPβ [24]. Beyond the BAF60 family, BRG1 (SMARCA4) modulates sterol metabolism through SREBP isoform‐specific interactions: while promoting SREBP2‐mediated cholesterogenesis via enhancer‐promoter looping [36], it concurrently represses SREBP1c‐driven fatty acid esterification through H3K9me3‐dependent chromatin compaction [16]. Since the accumulation and degeneration of lipids is one of the main characteristics of NAFLD [37], these pathways are especially crucial. These dual regulatory roles position BRG1 as a critical determinant of hepatic lipid accumulation in metabolic disorders.

Notably, *Cpt1a* is a rate‐limiting enzyme involved in mitochondrial β‐oxidation. BAF250a (ARID1A) safeguards mitochondrial FAO by maintaining chromatin accessibility at *Acox1* and *Cpt1a* promoters, with hepatocyte‐specific knockout mice developing age‐dependent steatohepatitis and HCC [38, 39, 40]. Mechanistically, BAF250a deficiency downregulates PPARα and insulin signalling pathways while leaving lipogenic transcription factors (e.g., SREBP1c, Fas) unaltered, indicating selective disruption of lipid catabolism over anabolism [40]. Collectively, SWI/SNF subunits coordinate lipid homeostasis through spatially and temporally distinct chromatin remodelling activities, offering therapeutic targets for metabolic liver diseases.

### 3.2 SWI/SNF Complex Governs Hepatic Immune Homeostasis Through Epigenetic‐Immunological Crosstalk

The liver, a critical hub for immune tolerance, requires precise spatiotemporal regulation of immunomodulatory gene expression. Central to this regulation, the SWI/SNF complex epigenetically coordinates innate and adaptive immune responses through chromatin remodelling. Mechanistically, BRG1 synergizes with STAT2 to amplify type I interferon (IFN‐α) pathway activation during viral infection, enhancing antiviral defence [41]. Parallel studies reveal BRG1‐mediated chromatin accessibility at B cell receptor loci drives lymphocyte proliferation and antibody diversification, with BRG1 deficiency impairing humoral immunity [42]. In T cell development, BRG1 orchestrates lineage commitment by facilitating the DN‐to‐DP (double negative to double positive) transition, as evidenced by thymocyte maturation arrest at the CD4^−^CD8^−^ stage in BRG1‐deficient mice [43]. Furthermore, BRG1 recruits AP‐1 transcription factors to activate hepatocyte‐derived CCL7 chemokine production, a process correlating with macrophage infiltration in human fibrotic livers [44].

Complementing these findings, ARID1A (BAF250a) inactivation in hepatocytes triggers steatohepatitis‐associated HCC with pronounced innate immune dysregulation. Hepatocyte‐specific *ARID1A* knockout mice exhibit F4/80^+^ macrophage and CD11c^+^ neutrophil infiltration in tumour margins, concomitant with IL‐6/TNF‐α hyperproduction and NF‐κB/STAT3 pathway activation [38]. This immunoinflammatory microenvironment accelerates hepatocarcinogenesis, establishing SWI/SNF dysfunction as a driver of tumour‐immune crosstalk. Collectively, SWI/SNF components emerge as epigenetic gatekeepers of hepatic immune equilibrium, balancing tolerance and inflammation through chromatin‐driven transcriptional control.

## 4 SWI/SNF Complex Orchestrates Liver Regeneration Through Epigenetic Coordination of Cell Cycle Dynamics

**FIGURE 3:**
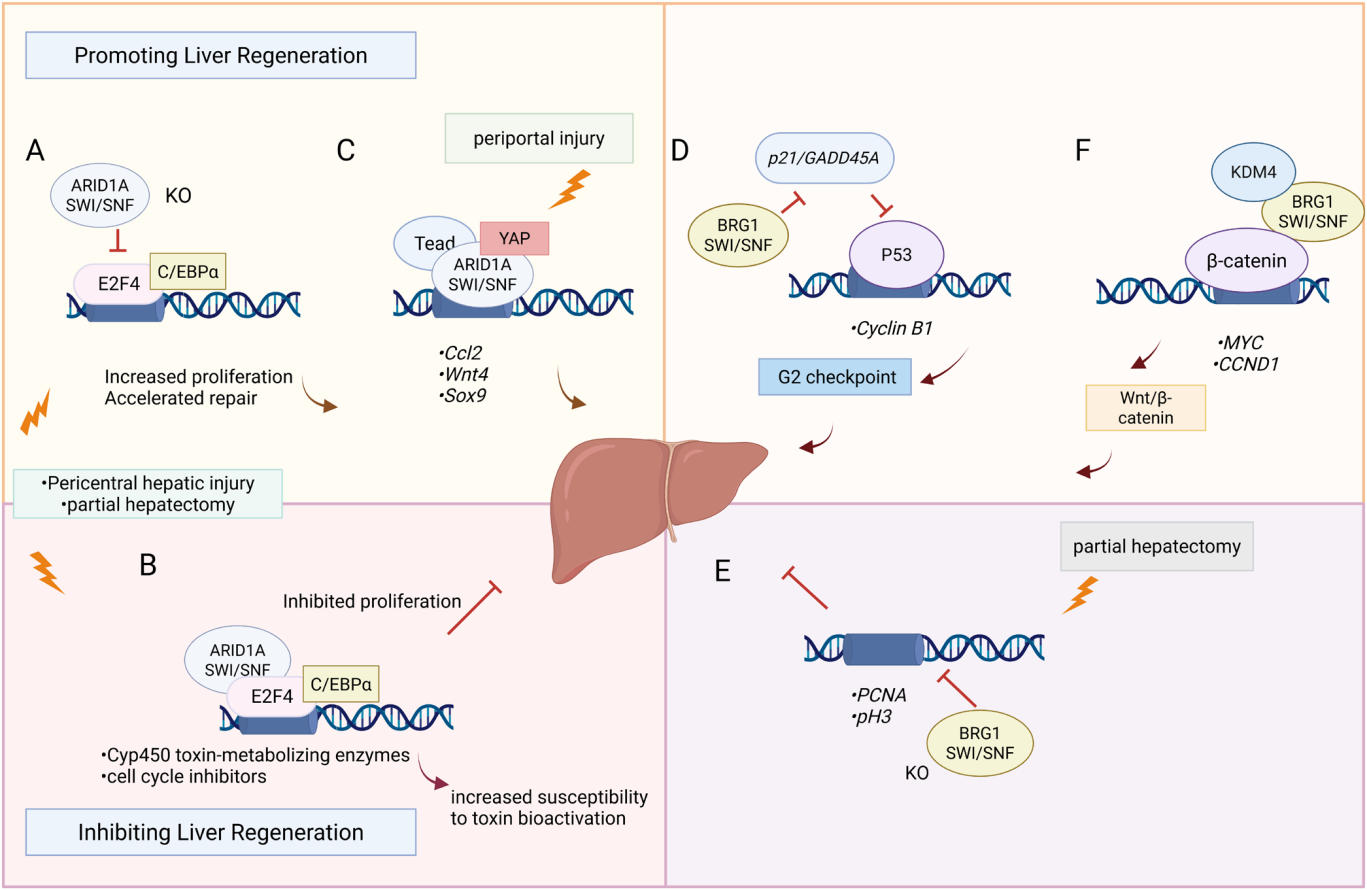
The SWI/SNF complex regulates liver regeneration. (A) ARID1A loss in pericentral injury/PH abrogates C/EBPα‐E2F4 repression, enhancing hepatocyte proliferation. (B) ARID1A recruits differentiation‐enforcing TFs and E2F4 to restrict proliferation and promote toxin susceptibility. (C) In periportal injury, ARID1A drives hepatocyte‐to‐LPLC transdifferentiation via YAP signalling. (D) BRG1 attenuates p53‐mediated cyclin B1 suppression to promote proliferation. (E) BRG1 depletion post‐PH reduces PCNA/pH 3 expression. (F) BRG1 recruits KDM4, binds β‐catenin and activates proliferation genes. GADD45A, growth arrest and DNA damage‐inducible protein 45 alpha; KDM4, lysine demethylase 4; p21, cyclin‐dependent kinase inhibitor; p53, tumour suppressor protein; YAP, yes‐associated protein.

The liver exhibits remarkable regenerative capacity to restore functional mass post‐injury, a process driven by cytokine and growth factor‐mediated signalling cascades [45]. Central to this regenerative response, the SWI/SNF complex epigenetically coordinates hepatocyte proliferation through its catalytic subunit BRG1 (Figure 3). BRG1 regulates cell cycle progression by modulating cyclin B1/CDK1 expression, with partial hepatectomy (PH) models demonstrating BRG1‐dependent chromatin remodelling peaks at post‐operative day 3 and resolution by day 7. However, when BRG1 was depleted, the expression of proliferating cell nuclear antigen (PCNA) and phosphorylated histone H3 (pH 3) decreased after PH [46]. Furthermore, at various phases of liver injury and regeneration, the interaction between BRG1 and BRM is essential for liver regeneration. Specifically, BRM plays a significant role in both the early stages of regeneration and the late stages of damage [47]. Mechanistically, BRG1 antagonises p53‐mediated cell cycle arrest by suppressing p21/GADD45A transcription, while concurrently enhancing Wnt/β‐catenin signalling through KDM4‐dependent activation of proliferative genes (e.g., *MYC*, *CCND1*) [48, 49]. This dual regulation balances proliferation checkpoints with regenerative demands. These findings indicate that BRG1 is required for hepatocyte proliferation in the restoration of liver tissue following PH.

As a core component of the SWI/SNF chromatin remodelling complex, ARID1A exhibits dual region‐specific roles. In periportal injuries, ARID1A establishes and maintains a permissive chromatin state (marked by H3K4me1/H3K27ac) at enhancers of hepatic progenitor cells (HPCs)‐associated genes (such as *Sox9* and *Opn*) in quiescent hepatocytes. This chromatin priming enables hepatocytes to respond to injury‐induced signals (e.g., the Hippo/YAP pathway), driving their transdifferentiation into HPCs to facilitate regeneration; ARID1A deficiency impairs YAP binding and consequently blocks HPCs formation [50]. Conversely, in pericentral injuries or PH models, ARID1A promotes the binding of differentiation‐enforcing transcription factors (C/EBPα, HNF4α) and the repressive factor E2F4 to target genes (e.g., *Cyp450* toxin‐metabolising enzymes and cell cycle inhibitors), thereby restricting proliferation and increasing susceptibility to toxin bioactivation. Loss of ARID1A remodels chromatin accessibility, abolishing transcriptional repression by C/EBPα and E2F4, which significantly enhances hepatocyte proliferative capacity and accelerates repair [51]. This region‐specific regulatory mechanism underscores ARID1A's role as a molecular switch that balances hepatocyte differentiation and dedifferentiation through spatiotemporal control of chromatin accessibility, ensuring the precision and adaptability of liver regeneration.

## 5 SWI/SNF Complex and Liver Disease

### 5.1 Epigenetic Dysregulation of SWI/SNF Complex Subunits in NAFLD Pathogenesis

NAFLD encompasses a spectrum ranging from simple steatosis (NAFL) to non‐alcoholic steatohepatitis (NASH), characterised by lobular inflammation, hepatocyte ballooning and perisinusoidal fibrosis, with increased risk of cirrhosis [52].

Mechanistic studies reveal ARID1A deficiency induces transcriptional reprogramming characterised by upregulated lipogenic pathways (e.g., SREBP1c activation) concurrent with suppressed β‐oxidation regulators, thereby accelerating steatotic progression. SREBP1c inhibition attenuates ARID1A‐mediated steatohepatitis, suggesting therapeutic potential for NASH management [39]. Complementary investigations identify BRG1 as a pro‐inflammatory epigenetic modulator that complexes with NF‐κB to stabilise chromatin accessibility at cytokine promoters (e.g., TNFα), amplifying feedforward inflammation correlating with human NASH severity [53, 54].

These findings with the observations in lipid metabolism regulation above‐mentioned collectively position SWI/SNF chromatin remodelers as pivotal regulators of NAFLD pathogenesis through dual mechanisms: disrupting lipid homeostasis via transcriptional imbalance between anabolic and catabolic pathways, while perpetuating sterile inflammation through cytokine‐driven microenvironment alterations. This epigenetic paradigm underscores the molecular complexity underlying disease progression from benign steatosis to inflammatory NASH phenotypes.

### 5.2 Epigenetic Regulation of SWI/SNF Complex Subunits in Hepatic Fibrosis

Chronic liver inflammation drives hepatic fibrosis, characterised by excessive deposition of cross‐linked collagen (predominantly types I and III) within the extracellular matrix. This fibrotic scarring progressively replaces functional parenchyma, causing architectural distortion and declining liver function [55].

The SWI/SNF complex participates in liver fibrosis regulation. As mentioned before, ARID1A plays a crucial role in regulating lipid synthesis and fatty acid oxidation in hepatocytes. ARID1A deletion disrupts lipid homeostasis, promoting NASH progression and pro‐inflammatory cytokine release that exacerbates fibrosis [39]. Additionally, the deletion of ARID1A also leads to the upregulation of a series of pro‐inflammatory cytokines, which are typically involved in liver inflammation and promote the fibrotic process. Fang et al. demonstrated that mice lacking ARID1A exhibited significant liver inflammation and injury after metabolic stress, accompanied by hepatocyte apoptosis and the occurrence of liver fibrosis [38]. This chronic inflammatory response not only affects the regenerative capacity of hepatocytes but may also activate hepatic stellate cells (HSCs), further promoting the development of liver fibrosis.

The TGF‐β/Smad signalling axis orchestrates HSC activation and extracellular matrix (ECM) homeostasis through transcriptional regulation of fibrogenic targets [56]. Smad3, a crucial protein in fibrotic signalling, binds directly to the DNA sequences of fibrotic genes (e.g., *Collagen* and *α‐SMA*) [57]. Experimental evidence reveals BRG1 as a critical co‐factor in this process. Carbon tetrachloride (CCl_4_)‐induced fibrotic murine models demonstrated BRG1 upregulation that potentiated Smad3 nuclear translocation and promoter recruitment at *α‐SMA* and *Col1a1* loci. Conversely, specific deletion of BRG1 decreased expression of *α‐SMA and Collagen I*, reduced collagen deposition and attenuated liver fibrosis in murine models. Meanwhile, pharmacological inhibition of BRG1 by PFI‐3, a specific bromodomain inhibitor of BRG1, led to the downregulation of pro‐fibrotic gene expression in immortalised HSC line LX‐2 [58, 59]. Consistently, BRG1 hyperactivation increased fibrogenic gene transcription and hastened fibrosis [53]. This demonstrates BRG1's indispensability in fibrotic progression, suggesting its therapeutic targeting could mitigate advanced liver fibrosis.

This mechanistic framework positions SWI/SNF components (BAF250a and BRG1) as epigenetic modulators of liver fibrosis through intersecting pathways: BAF250a maintains metabolic‐inflammation balance, while BRG1 mediates TGF‐β/Smad3‐driven ECM remodelling. Their dysregulation creates a permissive niche for HSC activation and progressive matrix deposition, suggesting therapeutic potential in targeting chromatin remodelling complexes.

### 5.3 Epigenetic Alterations of SWI/SNF Complex Submits in HCC

**FIGURE 4:**
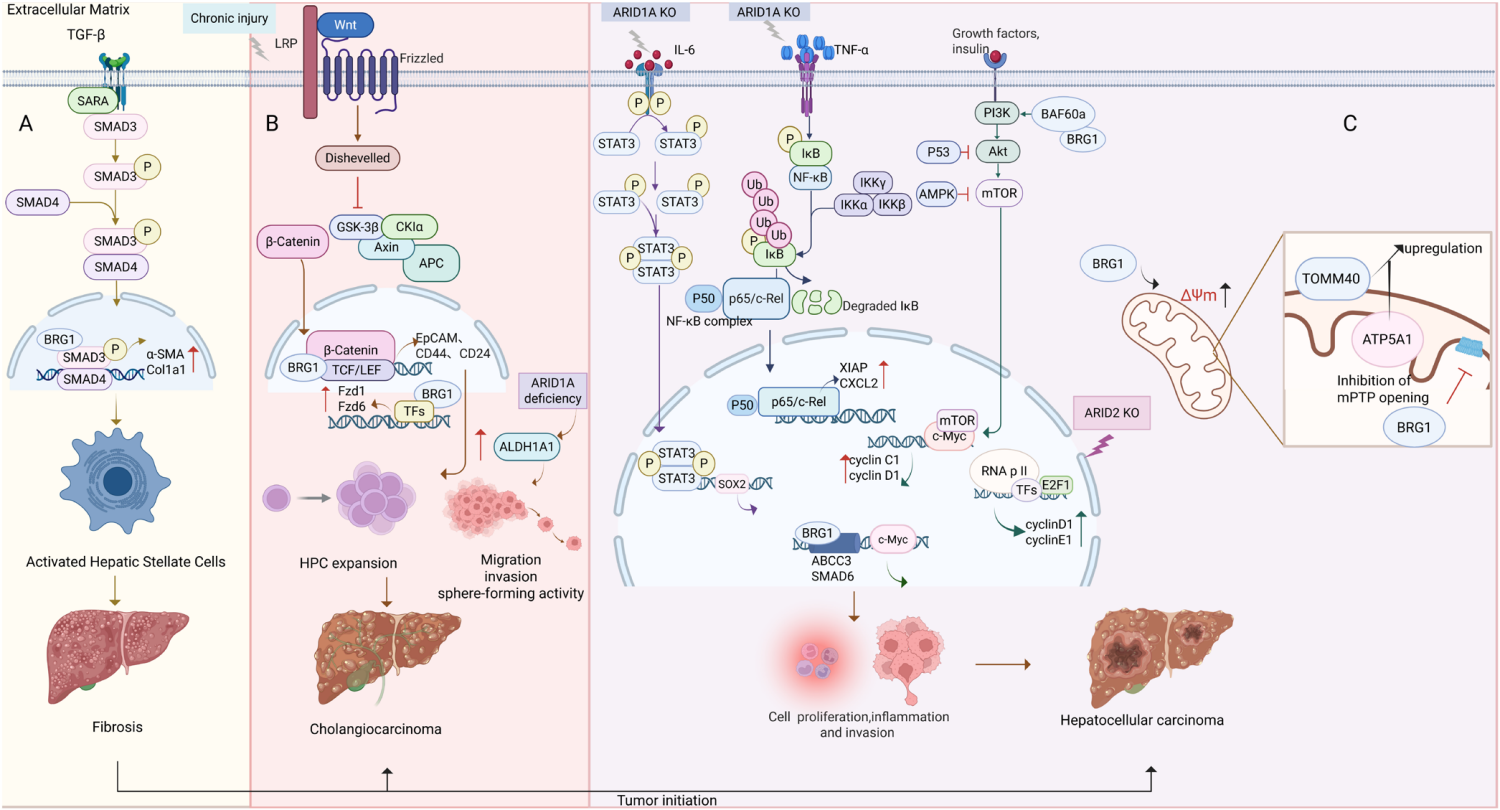
Role of SWI/SNF complexes and dysregulated signalling pathways in liver fibrosis and hepatobiliary carcinogenesis. (A) TGF‐β activates HSCs via SMAD3/4 phosphorylation, driving fibrosis. (B) Chronic injury and Wnt signalling stabilises β‐catenin, BRG1 cooperates with β‐catenin to expand HPCs, inducing cholangiocarcinoma phenotypes. (C) ARID1A‐KO amplifies IL‐6/STAT3 and TNF‐α/NF‐κB signalling, promoting tumour initiation. BRG1 enhances survival via mPTP inhibition and metabolic adaptation. BAF60a and BRG1 regulate the mTOR signalling pathway to promote Cyclin C1 and Cyclin D1 protein expression. MPTP, mitochondrial permeability transition pore; P, phosphorylation; SARA, SMAD anchor for receptor activation; TCF/LEF, T‐cell factor/lymphoid enhancer factor; TFs, transcription factors; Ub, ubiquitination. Red arrows denote upregulation.

HCC is the predominant form of primary liver malignancy. Chromatin remodelers like SWI/SNF serve as critical regulatory nodes (Figure 4). Approximately 25% of human malignancies harbour mutations in SWI/SNF subunit‐encoding genes [60]. Our prior transcriptomics revealed differential expression of SWI/SNF components between neoplastic and adjacent normal tissues via comparative transcriptomic analysis. Of particular significance, *SMARCD1* (encoding BAF60a) demonstrated marked overexpression in HCC specimens, mechanistically linked to mTOR pathway activation via upstream potentiation of PI3K/AKT signalling—a mechanism directly implicated in HCC [61].

ARID1A, a frequently mutated tumour suppressor [62], interacts with TOP2A to maintain mitotic fidelity. Its loss in HCC inactivates TGF‐β checkpoints, driving polyploidization, accelerating proliferation and metastasis [63]. Moreover, loss of E‐cadherin expression impairs intercellular adhesion and promotes cancer progression and metastasis. According to He et al., E‐cadherin levels were closely correlated with ARID1A expression, suggesting a role in migration and invasion [19]. Paradoxically, Zhao et al. identified a context‐dependent tumour‐suppressive role wherein ARID1A maintains genomic stability by modulating STAG1‐mediated telomere cohesion, suggesting its inactivation may paradoxically eliminate genomically unstable clones to reduce malignancy risk [64].

Emerging evidence complicates this paradigm. Sun et al.'s study offers fresh perspectives [65]. *ARID1A* is not just a tumour suppressor gene in HCC; it paradoxically promotes early hepatocarcinogenesis by enhancing *CYP450* (e.g., *Cyp2e1*)‐mediated ROS production. Furthermore, ARID1A promotes tumour growth in the early stages of the tumour, but after the tumour has established, its heterozygous and pure deletion speeds up the progression of HCC and metastases. This adds to our knowledge of the intricate involvement of ARID1A in the development of HCC and is in line with the function of tumour suppressor genes. Furthermore, there was a negative correlation between decreased ARID1A expression and nuclear expression of p53 or β‐catenin, indicating that ARID1A may stimulate HCC growth via distinct mechanisms. Hepatocyte‐specific ARID1A‐KO mice exhibited elevated steatohepatitis markers (TNF‐α, IL‐6) and STAT3/NF‐κB activation, fostering pro‐tumorigenic microenvironments [38]. Collectively, ARID1A functions as a spatiotemporal HCC modulator, with microenvironmental cues determining its tumour‐suppressive or ‐promoting switch. Elucidating this duality represents a critical research frontier.

Within the SWI/SNF chromatin remodelling complex, BAF250a and BAF250b are mutually exclusive paralogs (60% identity). While concurrent mutations in their encoding genes (ARID1A and ARID1B) may co‐occur in tumours, a synthetic lethal interaction between these paralogs has been identified, wherein ARID1B deficiency selectively eliminates ARID1A‐mutant cancer cells [66]. This epistatic relationship suggests that partial allelic loss of both genes may cooperatively drive tumorigenesis, while compensatory retention of at least one functional allele sustains oncogenic proliferation—a critical vulnerability for therapeutic exploitation.

ARID1B functions as a pleiotropic regulator of transcriptional programs and senescence pathways. Mechanistically, Tordella et al. demonstrated that ARID1B governs oncogene‐induced senescence (OIS) in hepatocarcinogenesis [67]. ARID1B deletion prevents OIS activation and synergizes with RAS signalling to accelerate liver tumorigenesis, implicating SWI/SNF‐mediated senescence regulation beyond canonical p16/p21 transcriptional control. Notably, ARID1B‐deficient cells exhibit dysregulated DNA damage response, oxidative stress modulation and impaired p53 activation. Furthermore, ectonucleoside triphosphate diphosphohydrolase 7 (ENTPD7) overexpression in ARID1B‐null contexts suppresses nucleotide biosynthesis, thereby bypassing senescence checkpoints. These findings not only expand the mechanistic repertoire of SWI/SNF complexes in hepatocarcinogenesis but also highlight the therapeutic potential of pro‐senescence agents targeting nucleotide metabolism in SWI/SNF‐mutant tumours.

Chronic HBV infection remains a predominant etiological factor for HCC [68], with the virally encoded X protein (HBx) serving as a critical mediator of both HBV replication and hepatocarcinogenesis [69, 70]. Chen et al. demonstrated that BAF155 overexpression potentiates HBx‐dependent transcriptional transactivation, a mechanism driving proto‐oncogene expression while paradoxically suppressing HCC cell proliferation [71]. Mechanistically, BAF155 stabilises HBx through direct protein interaction by competing with the 20S proteasome subunit PSMA7 to bind to HBx, shielding it from non‐ubiquitin‐dependent proteasomal degradation. These findings implicate BAF155 as a pivotal epigenetic cofactor in HBV‐associated HCC pathogenesis, functioning dually to amplify HBx oncogenicity while modulating its proteostatic regulation.

ARID2, encoding the BAF200 subunit exclusive to the PBAF complex, exhibits somatic mutations in 5%–8% of HCCs. Tumorigenic consequences of ARID2 dysregulation are multifactorial: (1) Transcriptomic profiling reveals significant ARID2 downregulation in HCC tissues, where it physically interacts with E2F1 to attenuate RNA Polymerase II recruitment at cyclin D1 (CCND1) and cyclin E1 (CCNE1) promoters. ARID2 deficiency precipitates G1/S phase acceleration via upregulated CCND1/CCNE1 expression, CDK4 activation and retinoblastoma (Rb) hyperphosphorylation; (2) ARID2 loss impairs nucleotide excision repair (NER) pathway fidelity, exacerbating DNA damage accumulation, mutagenic susceptibility and carcinogen sensitivity; (3) In HBV‐associated HCC, inverse correlation between ARID2 expression and HBx oncoprotein levels mechanistically links to HBx‐mediated suppression of ATOH1‐binding motifs in the ARID2 promoter—a regulatory axis potentiating viral hepatocarcinogenesis [72].

SMARCA4/BRG1 exhibits multifaceted oncogenic properties in HCC through mutational activation, transcriptional upregulation and epigenetic reprogramming [73]. Its overexpression drives tumour proliferation, invasion and chemoresistance via distinct molecular axes: (1) Transcriptional coactivation: BRG1 directly binds to promoters of proto‐oncogenes (e.g., ABCC3, SMAD6) [74, 75] and amplifies c‐MYC‐driven hepatocarcinogenesis, with conditional ablation in c‐MYC‐transgenic mice abolishing HCC development [76, 77]; (2) Lipid metabolic reprogramming: BRG1 suppresses glycosylated lysosomal membrane protein (GLMP) transcription, thereby inhibiting lysosomal lipid degradation and promoting lipid droplet accumulation. BRG1 deficiency activates the PIK3AP1/PI3K/AKT pathway through GLMP upregulation, reducing lipid storage while paradoxically enhancing pro‐survival signalling—a dual mechanism linking lipid metabolism to NAFLD‐HCC progression [78]. (3) Mitochondrial reprogramming: BRG1 enhances mitochondrial membrane potential (MMP) and suppresses mPTP opening by upregulating TOMM40/ATP5A1, thereby sustaining energy metabolism and apoptosis resistance [79]; (4) Stemness regulation: Through miR‐296‐5p/Sall4 axis, BRG1 activates stemness biomarkers and tumorsphere formation, correlating with postoperative recurrence [80]; (5) Inflammatory crosstalk: BRG1 recruits lncRNA MALAT1 to NF‐κB targets (IL‐6/CXCL8), amplifying inflammation‐driven HCC progression [81]; (6) Clinically, BRG1 overexpression predicts aggressive phenotypes and poor prognosis, with nuclear localization serving as an early recurrence biomarker. Its functional duality—as both SWI/SNF chromatin remodeler and metabolic/transcriptional amplifier—positions BRG1 as a central epigenetic hub coordinating proliferation, survival and microenvironment adaptation. Therapeutic strategies targeting BRG1‐dependent pathways (ABCC3 inhibition, MALAT1 silencing, or GLMP/PI3K‐AKT modulation) demonstrate preclinical efficacy, validating its role as an actionable node in HCC pathogenesis [73, 76, 77].

While SMARCA2 exhibits low mutational frequency in malignancies, its transcriptional repression is a recurrent feature across multiple cancer cell lines and primary tumours. Emerging evidence reveals a functional antagonism between SMARCA2 (BRM) and SMARCA4 (BRG1) in oncogenic progression. Computational meta‐analysis by Jose et al. delineated divergent prognostic associations: SMARCA4 overexpression correlates with tumour invasiveness, whereas elevated SMARCA2 expression marks benign differentiation states—a dichotomy particularly pronounced in HCC [21]. This antagonistic relationship is mechanistically rooted in SWI/SNF complex dependency; SMARCA4‐mutant malignancies exhibit synthetic lethal vulnerability to SMARCA2 ablation, as residual complexes rely exclusively on SMARCA2‐derived ATPase activity [82].

Concurrently, SNF5 (SMARCB1), a core SWI/SNF subunit regulating proliferation‐apoptosis balance [83], demonstrates tumour‐suppressive functions in HCC. Clinically, SNF5 downregulation in HCC tissues correlates with advanced tumour grade, reduced sorafenib sensitivity and diminished overall survival [84]. Mechanistically, SNF5 depletion potentiates TGF‐β1‐driven oncogenic dedifferentiation while attenuating chemotherapeutic response. These findings collectively nominate SNF5 as both a prognostic biomarker and therapeutic target in HCC management.

### 5.4 Epigenetic Modifications of SWI/SNF Complex Submits in CCA

CCA is the second most prevalent primary hepatic malignancy. In recent years, the regulatory role of the SWI/SNF complex in CCA has received increasing attention.

BRG1 is strongly upregulated in intrahepatic cholangiocarcinoma (iCCA) tissues. It is well known that iCCA originates from the expansion of HPCs, which are always accompanied by peritubular cirrhosis [85]. In our previous study, we systematically investigated the role of BRG1 in HPC expansion, liver fibrosis and iCCA development [86]. And we found BRG1 was levated in HPCs after CCl_4_ or thioacetamide (TAA) exposure, and ablation of BRG1 dramatically attenuated HPC expansion and liver fibrosis in different models. Mechanistically, BRG1 regulates cell growth by activating Wnt/β‐catenin signalling, which is characterised by a certain concentration of β‐catenin in the cytoplasm that is transported into the nucleus with the help of factors like Rac1, which has been shown to be necessary for stem cell maintenance, HPC expansion and the development of HCC [87]. On the contrary, inhibition of BRG1 only in HPC can disrupt Wnt/β‐catenin signalling, effectively inhibit HPC expansion, improve liver histological features and finally slow down the growth process of iCCA development [86].

Recent studies have shown that the clinicopathologic features of CCA are associated with BAF250a abnormalities [88]. Notably, consistent with the function of ARID1A in other cancer types (including gastric, lung and renal cancers), it acts as a tumour suppressor gene in CCA [89]. ARID1A and p53 have been reported to cooperate to prevent tumorigenesis by transcriptionally activating downstream genes of tumour suppressors [90]. According to Chan‐on et al., in the pathophysiology of CCA, the proliferation of CCA cells is greatly increased when ARID1A is silenced; in contrast, the cell proliferation process is disrupted when ARID1A expression is up‐regulated [91]. Yoshino et al. also discovered that changes in ARID1A caused CCA cells to upregulate a number of genes, including *Aldh1a1*, a hallmark of cancer stem cells. ARID1A knockdown increases cell migration, invasion and sphere‐forming activity in CCA cell lines; these effects may be strongly linked to transcriptional suppression of ALDH1A1 expression and decreased histone H3K27 acetylation [22]. According to the research conducted by Sasaki et al., the development of CCA may begin with the loss of ARID1A expression. It describes the establishment of a unique molecular mechanism associated with amorphous and tubular adenocarcinomas. This precursor lesion of ARID1A expression loss may cause a precancerous CCA [88].

### 5.5 Epigenetic Impact of SWI/SNF Complex Submits in Ischemia–Reperfusion Injury (IRI)

Liver ischemia–reperfusion (I/R) are separated into two phases, ischemic and reperfusion, during which oxidative stress, inflammation and mitochondrial dysfunction occur [92], resulting in serious harm. It is a common complication in liver procedures, including liver transplantation, as well as resection and abdominal trauma. RNA sequencing research revealed that NOXA (encoded by the *Pmaip1* gene) is a new target of BRG1, a known apoptosis mediator. BRG1 improves hepatocyte apoptotic sensitivity by increasing NOXA expression. And increased NOXA expression can interact with anti‐apoptotic proteins such as Bcl‐2 or Bcl‐xL, releasing cytochromogranin C and triggering the cascade response, resulting in hepatocyte apoptosis [93]. This implies that BRG1 can alter hepatocyte apoptosis, resulting in liver I/R damage. However, the SWI/SNF complex counteracts this damage through BRG1‐mediated activation of the Nrf2 antioxidant pathway. Mechanistically, BRG1 forms a stable complex with Nrf2 to enhance chromatin accessibility at antioxidant response element (ARE)‐containing promoters, including NQO1 and HO‐1, thereby potentiating transcriptional activation of cytoprotective genes and preserving redox equilibrium [94].

## 6 Targeted Therapeutic Potential of the SWI/SNF Complex

Synthetic lethality targeting SWI/SNF subunits holds therapeutic potential for SWI/SNF‐mutant malignancies. Bromodomain‐containing ATPases (e.g., SMARCA2/4) offer dual targeting domains (bromodomain and ATPase) [95]. While bromodomain inhibitors such as JQ1 (targeting BRD4) demonstrate preclinical efficacy [96], selective targeting remains challenging due to structural homology between SMARCA2 and SMARCA4–a critical barrier to developing isoform‐specific inhibitors.

Emerging strategies exploit interdependencies within SWI/SNF paralogs. ARID1A‐ARID1B synthetic lethality provides a therapeutic window in ARID1A‐mutant cancers, analogous to EZH2‐PRC2 disruption via peptide‐mediated interference [97]. This approach could be adapted to destabilise ARID1B‐containing SWI/SNF complexes. Furthermore, ARID1B's non‐canonical role in histone H2B monoubiquitination via E3 ligase activity introduces an orthogonal targeting axis [98]. Although the mechanistic contribution of this activity to synthetic lethality requires elucidation, pharmacological inhibition of ARID1B's ubiquitination function may synergize with existing epigenetic therapies.

PFI‐3, a selective pharmacological antagonist targeting the bromodomain of the SWI/SNF chromatin remodelling complex, disrupts chromatin remodelling and DNA damage repair, thereby inducing cancer cell death through necrosis and senescence. PFI‐3's effect on gene expression is tightly linked to the regulation of SWI/SNF, and it depends on whether cancer cells need SWI/SNF for DNA repair [99]. As mentioned, we demonstrated that BRG1 inhibition represents a promising therapeutic strategy for mitigating HPC‐driven pathologies, including cirrhosis and iCCA [86]. In our experimental model, PFI‐3 was employed to disrupt SWI/SNF‐mediated chromatin remodelling. PFI‐3 treatment significantly attenuated HPC expansion, as evidenced by reduced β‐catenin nuclear translocation and EpCAM expression. Notably, BRG1 inhibition in vivo resulted in pronounced phenotypic improvements. These findings validate BRG1 as a tractable epigenetic target for intercepting premalignant hepatobiliary. However, the library of compounds screened for PF3‐1 has limitations and is too small in number and variety. More research is still needed to develop more specifically targeted inhibitors.

## 7 Conclusion and Outlook

The SWI/SNF chromatin remodelling complex serves as a critical epigenetic modulator in hepatic physiology and pathobiology (Table 1). Emerging therapeutic paradigms targeting this complex—including precision epigenetic modulation, rational immunotherapy combinations and pro‐regenerative strategies—are poised to revolutionise the management of liver diseases. Concurrently, the integration of CRISPR‐based gene editing with patient‐specific multi‐omics profiling will enable tailored therapeutic interventions, accelerating clinical translation of SWI/SNF‐focused modalities.

**TABLE 1:** Dysregulation of SWI/SNF complex subunits in liver diseases.

Submit	Isoform	Gene	Regulation/disease	Expression regulation and pathological correlation
SNF5		*SMARCB1*	Development [17], HCC [84]	Inactivating mutation: Disruption of intercellular junctions, impaired hepatocyte formation, increased cell proliferation Low expression: TGF‐β1 expression is upregulated to promote cell growth and migration, tumour proliferation
BAF60	BAF60a BAF60b BAF60c	*SMARCD1* *SMARCD2* *SMARCD3*	Lipid metabolism [29, 30, 32, 33], HCC [61] Lipid metabolism [29, 31, 34, 35]	Low expression: Impairs fatty acid oxidation, leading to hepatic steatosis after fasting High expression: Diet‐induced hypercholesterolemia and atherosclerosis
				Low expression: Lipid accumulation
				High expression: Promotes insulin‐induced lipogenesis, increasing triglyceride levels
BRM		*SMARCA2*	HCC [21], regeneration [47]	High expression: Benign differentiation of the tumour; may predominate at the end of the injury phase and the beginning of the regeneration phase
BRG1		*SMARCA4*	Lipid metabolism [16, 36], immunity [41, 42, 43, 44], regeneration [46, 47, 49], hepatic fibrosis [53, 54, 58, 59], HCC [73, 74, 75, 76, 77, 78, 79, 80, 81], CCA [86], IRI [93, 94]	High expression: Lipid accumulation; the improvement of immune function; Expansion of HPCs Low expression: Regeneration damage; tumour proliferation and invasion; collagen deposition
BAF200		*ARID2*	HCC [72]	Inactivating mutation: Associated with the four major subtypes of HCC
BAF250	BAF250a	*ARID1A*	Regeneration [50, 51], immunity [38], lipid metabolism [39], hepatic fibrosis [38], HCC [19, 38, 64, 65] CCA [22, 88]	Inactivating mutation: Dysregulated lipid metabolism, steatosis and inflammation; cells re‐enter the cell cycle; pronounced innate immune dysregulation; tumour invasion and metastasis
	BAF250b	*ARID1B*	HCC [66, 67]	Inactivating mutation: tumour development; preventing the occurrence of OIS
BAF155		*SMARCC1*	HCC [71]	High expression: Maintains the stability of HBx, promoting the occurrence of HCC

Abbreviations: CCA, cholangiocarcinoma; HCC, hepatocellular carcinoma; HPCs, hepatic progenitor cells; IRI, ischemia–reperfusion injury; OIS, oncogene‐induced senescence.

Three critical knowledge gaps persist despite advances:

- Isoform‐specific functionality: The spatiotemporal regulation of SWI/SNF variants (cBAF, PBAF, ncBAF) across liver disease states remains incompletely characterised, particularly their dynamic recruitment to chromatin under pharmacological perturbation.
- Interaction networks: Systematic mapping of SWI/SNF interplay with hepatic transcription factors (e.g., HNF4α, FOXA2) and co‐regulators is essential to uncover disease‐specific epigenetic circuitry.
- Metabolic crosstalk: The complex's role in coordinating liver regeneration and metabolic homeostasis, especially in NAFLD‐HCC transition, requires mechanistic elucidation through lineage‐tracing models.

Technological innovations provide unprecedented resolution for these investigations. CRISPR‐Cas9 genome editing platforms permit subunit‐specific knockout/knock‐in to dissect isoform contributions; single‐cell multi‐omics can delineate SWI/SNF‐mediated cellular heterogeneity; and chromatin accessibility mapping (ATAC‐seq) coupled with ChIP‐seq will resolve context‐dependent chromatin remodelling dynamics. These approaches collectively may advance our understanding of SWI/SNF as a molecular rheostat governing hepatic pathology and repair.

## Author Contributions

**Rui Li**, **Bo Zhang** and **Yongjie Zhou:** writing original draft, conceptualization, visualisation. **Xinyu Wei:** editing and conceptualization. **Yongjie Zhou** and **Jiayin Yang:** writing‐review and editing, conceptualization, supervision and funding acquisition.

## Funding

This work was supported by the National Natural Science Foundation of China (No. 82173255), the National Science and Technology Major Project (No. 2023ZD0502400), and 1.3.5 project for disciplines of excellence from West China Hospital of Sichuan University (No. ZYGD24002).

## Conflicts of Interest

The authors declare no conflicts of interest.

## Data Availability

Data sharing not applicable to this article as no datasets were generated or analysed during the current study.
